# Poorer White Matter Microstructure Predicts Slower and More Variable Reaction Time Performance: Evidence for a Neural Noise Hypothesis in a Large Lifespan Cohort

**DOI:** 10.1523/JNEUROSCI.1042-22.2023

**Published:** 2023-05-10

**Authors:** Ethan M. McCormick, Cambridge Centre for Ageing and Neuroscience, Rogier A. Kievit

**Affiliations:** ^1^Cognitive Neuroscience Department, Donders Institute for Brain, Cognition and Behavior, Radboud University Medical Center, 6525 GD Nijmegen, The Netherlands; ^2^Methodology and Statistics Department, Institute of Psychology, Leiden University, 2333 AK Leiden, The Netherlands; ^3^Department of Psychology and Neuroscience, University of North Carolina at Chapel Hill, North Carolina, 27599; ^4^MRC Cognition and Brain Sciences Unit, University of Cambridge, Cambridge CB2 7EF, United Kingdom

**Keywords:** aging, dynamic structural equation modeling, lifespan, reaction time, white matter

## Abstract

Most prior research has focused on characterizing averages in cognition, brain characteristics, or behavior, and attempting to predict differences in these averages among individuals. However, this overwhelming focus on mean levels may leave us with an incomplete picture of what drives individual differences in behavioral phenotypes by ignoring the variability of behavior around an individual's mean. In particular, enhanced white matter (WM) structural microstructure has been hypothesized to support consistent behavioral performance by decreasing Gaussian noise in signal transfer. Conversely, lower indices of WM microstructure are associated with greater within-subject variance in the ability to deploy performance-related resources, especially in clinical populations. We tested a mechanistic account of the “neural noise” hypothesis in a large adult lifespan cohort (Cambridge Centre for Ageing and Neuroscience) with over 2500 adults (ages 18-102; 1508 female; 1173 male; 2681 behavioral sessions; 708 MRI scans) using WM fractional anisotropy to predict mean levels and variability in reaction time performance on a simple behavioral task using a dynamic structural equation model. By modeling robust and reliable individual differences in within-person variability, we found support for a neural noise hypothesis (Kail, 1997), with lower fractional anisotropy predicted individual differences in separable components of behavioral performance estimated using dynamic structural equation model, including slower mean responses and increased variability. These effects remained when including age, suggesting consistent effects of WM microstructure across the adult lifespan unique from concurrent effects of aging. Crucially, we show that variability can be reliably separated from mean performance using advanced modeling tools, enabling tests of distinct hypotheses for each component of performance.

**SIGNIFICANCE STATEMENT** Human cognitive performance is defined not just by the long-run average, but trial-to-trial variability around that average. However, investigations of cognitive abilities and changes during aging have largely ignored this variability component of behavior. We provide evidence that white matter (WM) microstructure predicts individual differences in mean performance and variability in a sample spanning the adult lifespan (18-102). Unlike prior studies of cognitive performance and variability, we modeled variability directly and distinct from mean performance using a dynamic structural equation model, which allows us to decouple variability from mean performance and other complex features of performance (e.g., autoregression). The effects of WM were robust above the effect of age, highlighting the role of WM in promoting fast and consistent performance.

## Introduction

Prominent theories of cognitive aging posit a central role for reductions in mental processing speed during later life (Salthouse, 1996). These theories focus primarily on decreases in mean performance (i.e., slowing-down) but mostly ignore variability in processing speed, despite a long acknowledgment that variability around the mean provides unique information (Woodrow, 1932; Fiske and Rice, 1955; Nesselroade, 1991). Prior work shows that average reaction time (RT) displays steep improvement early in life, followed by gradual slowing into aging (Li et al., 2004). Complementary work shows a similar pattern for behavioral variability (Williams et al., 2005). Crucially, despite these similar patterns, evidence strongly suggests that variability provides unique insight into cognitive aging above and beyond mean performance alone (Eizenman et al., 1997; Dykiert et al., 2012; Gamaldo et al., 2012). For instance, trial-to-trial variability might reflect different strategies, momentary lapses (Adam et al., 2015), or endogenous differences in the signal-to-noise ratio of neural-evoked responses.

The neural noise hypothesis (Kail, 1997) posits that the signal-to-noise ratio of the CNS degrades during aging, leading to deficits in processing speed. Electrophysiological recordings have shown that increased neural noise (i.e., desynchronized neural oscillations) led to slower behavioral performance in older adults (Voytek et al., 2015; Dave et al., 2018) because of disrupted long-range communication and synchronization between neural regions (Voytek and Knight, 2015). Reduced inter-region communication suggests a causal role for demyelination of neuronal axons carrying signals between cortical regions (Peters, 2009). While the mechanisms of progressive demyelination leading to slowing of RT are debated (Peters, 2009; Bartzokis et al., 2010), this relationship is robust in normative samples (Turken et al., 2008; Fjell et al., 2011; Tamnes et al., 2012; Bennett and Madden, 2014). Additionally, in clinical samples with specific focal damage to white matter (WM) (e.g., multiple sclerosis), increased WM damage is associated with increased behavioral variability (Britton et al., 1991; Bunce et al., 2007). While prior findings have focused primarily on mean processing speed (but see Li and Lindenberger, 1999), this logic extends naturally to variability (MacDonald et al., 2006; Halliday et al., 2019; Sorg et al., 2021). Impaired WM is more prone to random leakage from axonal signals that lead to inconsistencies in behavioral performance. As such, we hypothesize that reduced WM measures should predict slower overall performance and increased trial-to-trial variability.

A key limitation in prior work on variability is the widespread use of measures, such as the SD of response times (iSD) or coefficient of variation (iCV; equal to the iSD divided by the mean) across trials (e.g., Haynes et al., 2017). Unfortunately, these simple estimates of variability fail to account for complex features of behavioral performance (e.g., trends or autoregression), leading to systematic overestimates or underestimates of variability (Wang and Grimm, 2012). To address these challenges, we used dynamic structural equation modeling (DSEM) (Asparouhov et al., 2018), which combines strengths of time-series analysis with hierarchical random effects in a structural equation framework. This approach allows us to test trial-to-trial predictors of RT data at the within-person level, and explain person-to-person differences in mean RT and variability simultaneously (McNeish and Hamaker, 2020). Using this innovative framework for capturing variability deconfounds sources of variance that might otherwise bias individual differences performance features, and allows us to test different causal mechanisms associated with individual differences in mean and variability in RT.

We tested this neural noise hypothesis in a large cohort of adults (age 18-102) (Shafto et al., 2014) who completed a set of RT tasks (*N* = 2681) and underwent a diffusion-weighted scan (*N* = 708). We modeled individual differences in four aspects of behavioral performance: mean RT, variability, trends across the task, and the autoregressive effect between adjacent trials. We incorporated differences in WM and age to examine how they predicted each component of processing speed performance. We hypothesized that reduced WM microstructure would predict increased mean RT and heightened variability (i.e., slower, less consistent performance), and that these effects would be robust to including age in the model. In doing so, we pursued dual goals: (1) to conduct a theoretical test of the neural noise hypothesis and (2) to use that test to outline a generative DSEM framework for testing hypotheses related to variability for future work in this area.

## Materials and Methods

For consistency and precision, portions of the text in this section are drawn from prior work describing the Cambridge Center for Ageing and Neuroscience (Cam-CAN) dataset (Shafto et al., 2014; Taylor et al., 2017).

### Participants

Data were provided by healthy adult participants in two phases of data collection. Stage I consisted of at-home interviews of individuals living in the Cambridge City, UK region. During Stage II, a subset of these participants was invited to complete a neuroimaging session, where structural and functional scans were obtained. Participants at Stage II were recruited in deciles between 18 and 87 years of age, with a goal of equal sample sizes (∼100) at each decile. In the current analyses, we used behavioral data (“simple” response time [SRT]) from 2681 healthy adults (1508 female; 1173 male) from Stage I and additional behavioral data (visual short-term memory [VSTM]) and measures of structural WM from 708 adults (359 female; 349 male) from Stage II. A subset of these data has been analyzed previously (Kievit et al., 2016) using more simplistic methods. Exclusion criteria included MR safety contraindications (e.g., pacemakers), learning disability (living at home), cognitive impairment (Mini-Mental State Examination score of ≤24) (Folstein et al., 1975), and reduced response from individuals with limited longstanding illness or disability (for full information on exclusion criteria, see Shafto et al., 2014, their Table 1). Before the interview, individuals gave written informed consent for the study and record linkage. Individuals who lack the capacity to give consent were not included. Written informed consent was also given by participants at each session for Stages II and III (Shafto et al., 2014).

### Experimental design

#### Behavioral tasks

The description of the behavioral tasks is copied, in part, from Shafto et al. (2014). For additional information regarding the tasks and requests for data access, see the Cam-CAN dataset inventory (https://camcan-archive.mrc-cbu.cam.ac.uk/dataaccess/).

##### SRT task

An SRT task was used to assess basic aspects of speeded responses. In the SRT, participants viewed an image of a hand with blank circles above each finger, while resting their right hand on a response box with four buttons, one for each finger. When a given circle turned black on the image, the participants were instructed to press with the relevant finger as quickly as possible. On pressing the button (or after a maximum of 3 s), the circle became blank again and the variable intertrial interval began. The intertrial interval was varied pseudo-randomly with a positively skewed distribution (mean = 3.7 s, median = 3.9 s, range = 1.8-6.8 s). The SRT consisted of 50 trials, and the principal outcome measure was RT from stimulus onset to button press. For a visualization of the task, see Kievit et al. (2016, their Fig. 2a).

##### Visual short term memory task

This task assesses the processes underpinning VSTM. In the VSTM, participants viewed a series of circular disks presented briefly on a computer screen. After a brief delay, participants reported the color of a cued disk, by selecting from a color wheel that displays a rainbow of hues. On each trial, participants saw a display for 250 ms which contains a central fixation and one to four colored disks, with the colors chosen at random. The locations of the disks on the screen were randomly selected from eight points equidistant from a central fixation. Following the brief encoding display, there was a 900 ms blank screen, and then one of the disk locations was highlighted with a border and the response color wheel appeared. On half of trials, any uncued disks also reappeared, to provide the context within which the disk was encoded. Participants indicated their confidence in the selected color by the length of time they held down their finger: as they held their finger down for longer, white CIs spread out around the selected point indicating more uncertainty about their selection. The response interval did not have a time limit: after participants confirmed their response, there was an 830 ms fixation period before the next trial began. After a brief practice period, participants completed two blocks of 112 trials, with set-size and probe context being counterbalanced and randomly intermixed within each. Participants also completed a perceptual control block of 56 trials, where single disks were presented at fixation along with the color wheel, until the participant matched their hue by selecting the appropriate point on the surrounding wheel. There were two possible responses to model with the VSTM data: a first response and a final response. In general, we discuss results related to the final response, but full results from both models are available (https://osf.io/nkjdt/). We note major differences if relevant.

#### WM microstructure

WM microstructure was assessed using the fractional anisotropy (FA) of WM tracts derived from diffusion weighted imaging; see prior work for full details on the relevant scan parameters (Taylor et al., 2017) and processing pipeline (Kievit et al., 2016). We used FA given its widespread use in investigations of aging and WM; however, it should be noted that FA is a complex measure and its exact relation to WM health is not fully understood (Jones et al., 2013). Furthermore, its relatively simple model structure may not capture especially complex features of WM organization (e.g., crossing fiber bundles). Consistent with prior work, mean FA was computed for the 10 tracts defined by the Johns Hopkins University WM tractography atlas (Hua et al., 2008; for tract visualization, see Kievit et al., 2016): the anterior thalamic radiations (ATRs), corticospinal tract, dorsal cingulate gyrus, ventral cingulate gyrus (CINGHipp), forceps major, forceps minor, inferior fronto-occipital fasciculus, inferior longitudinal fasciculus, superior longitudinal fasciculus, and uncinate fasciculus. As a sensitivity analysis, FA values >4 SDs from the mean in each tract (0.27% of values) were excluded for the core set of analyses. Model results were not substantively impacted, and so all values were retained in the presented results.

We faced a challenge when using the different WM tracts as predictors in the DSEM (detailed below) because of the high correlations across measures (54% of the total variance explained by the first principal component). We considered several options, but each came with limitations: (1) principal component analysis-derived scores did not allow for missing data; (2) WM tracts do not theoretically conform to a reflective latent variable structure; and (3) including all tracts as simultaneous predictors resulted in unstable results because of the high multicollinearity (Lavery et al., 2019). Furthermore, prior work (de Mooij et al., 2018) showed that, while WM tracts are correlated, they do not show a unidimensional or simple factor structure (i.e., predominant loading onto a single factor, negative factor loadings, etc.). As such, we fit models for each tract separately and looked across models for consistent findings. We thereby present a single tract (the ATR) as a canonical example, while noting any deviation from the canonical pattern of effects if needed. We do not attempt to draw specific theoretical inferences for individual tracts, but rather take them as repeated instances of a WM general effect. Consistent effects across tracts would indicate that the effects are generally related to WM FA, while inconsistent effects might suggest functional specificity, which we do not expect.

### Statistical analysis

#### DSEM

**Figure 1.**
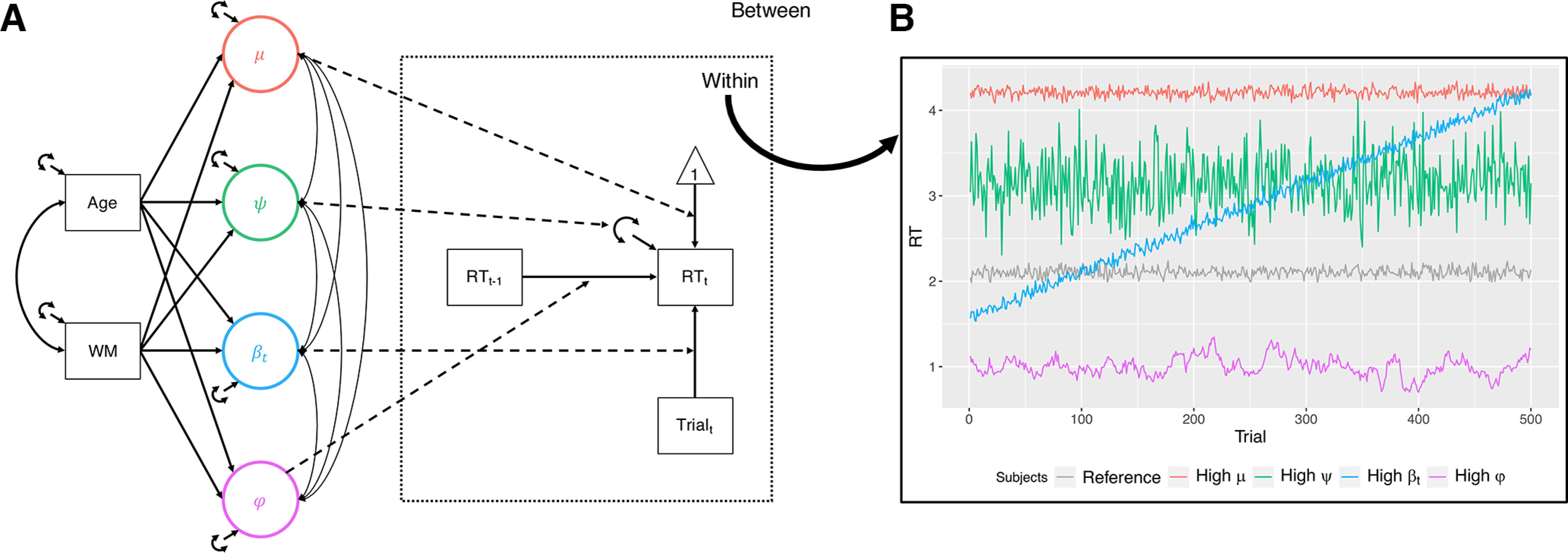
***A***, Model schematic. We estimated four random effects (μ, ψ, β*_t_*, and φ) at the between-person level from the within-person model (dashed lines) of RT (RT*_t_*) regressed on its prior value (RT_t-1_) and the current trial number (Trial_t_). Observed variables are depicted in boxes and latent variables in circles, as is conventional. The random effects were regressed (solid single-headed arrows) on the covariates, Age and WM FA (tracts were modeled individually so the identity of this covariate varied over models). Finally, the covariance between Age and FA (solid double-headed arrow, left) and residual covariances between the random effects (solid double-headed arrows, right) were estimated. ***B***, Exemplar time-series. We simulated exemplar time-series to highlight the impact of higher values on each of the four random effects. The exemplars differed from the reference time-series (gray) in mean (red; High μ), variance (green; High ψ), trend (blue; High β*_t_*), and autoregressive (purple; High φ) parameters.

All models were fit using the DSEM module in M*plus* version 8 for intensive longitudinal data (Asparouhov et al., 2018). DSEM is a flexible modeling framework, which combines strengths of traditional time-series analyses, multilevel models, and structural equation models (for a primer on DSEM, see McNeish and Hamaker, 2020). DSEM also allows for the simultaneous estimation of the random effects of behavioral performance and their association with covariates of interest, rather than a 2 step approach that does not propagate the uncertainty in estimates across levels of analysis (Wang and Grimm, 2012). At the within-person trial level, we modeled the natural log of RT (logRT) for both SRT and VSTM behavior on a given trial as a function of RT at the prior trial (φ) and the trial number (β*_t_*). Phi (φ) represents the autoregressive effect, or inertia of performance between adjacent trials. Individuals with high autoregressive effects would show slower cycles of faster and slower performance around their mean, while smaller autoregressive effects would reflect larger differences in performance between adjacent trials (compare the gray and pink exemplar time-series in Fig. 1*B*). The trend (β*_t_*) parameter reflects overall gains or losses in performance across the task session (here the trend across trials, but this could reflect other metrics of time; McCormick, 2021) (compare the gray and blue exemplar time-series in Fig. 1*B*). At the person level, we modeled random effects for φ and the trend (β*_t_*), as well as for the mean (μ) and variance (ψ) of RT across the task. By default, the between-person ψ parameter was estimated with a log-linear model to aid in estimation (McNeish and Hamaker, 2020). For each task, we compared a fixed-effects model to one where each of these parameters was allowed to vary freely. To assess the robustness of the results and compare models, we took several steps to evaluate the results. Model convergence was achieved when the potential scale reduction (PSR) metric, which estimates the potential reduction in the width of the posterior parameter distribution with infinite subsequent draws, was <1.10 (Gelman and Rubin, 1992), reflecting <10% potential narrowing compared the current distribution. Trace plots associated with the estimated parameter were examined visually to ensure that chains converged to a random scatter around a fixed mean (i.e., flat traces). To ensure model robustness, we compared a solution with 10,000 iterations to one with 20,000 iterations using the same random seed. Parameter changes between models were assessed with a measure of relative bias (i.e., the difference between the two parameter solutions divided by the original parameter). We then estimated the same model with 20,000 iterations twice more, with different random seeds to assess the impact of starting values on the final solution. If bias was low (<5%) across solutions, we retained the model; otherwise, we doubled the iterations for all solutions and assessed relative bias on the new models. All models presented here converged to acceptable values (for all model diagnostics and comparisons, see the analysis code; https://osf.io/nkjdt/). To correct for the multiple models, we applied false discovery rate correction to the significance tests. Because the standard *Mplus* output only gives floating point precision to the third decimal place, we calculated the adjusted *p* values from the estimate and posterior SE.

We then built a series of conditional models predicting the random effects by including the WM tract FA (with the ATR as the canonical example) and age separately before including both in a final conditional model. The unconditional model can be expressed in the multilevel equation below, for person *i* at trial *t*:

Level 1:

$$\log\left({\mathrm{RT}}_{t,i}\right)=\mu_{i}+\varphi_{i}{\mathrm{RT}}_{t-1,i}+\beta_{\mathrm{ti}}{\mathrm{Trial}}_{t,1}+\varepsilon_{t,i}\varepsilon_{t,i}∼N\left(0,\psi_{i}\right)$$

Level 2:

(1) $$\begin{array}{c} \mu_{i}=\gamma_{00}+u_{0i}\\ \varphi_{i}=\gamma_{10}+u_{1i}\\ \beta_{ti}=\gamma_{20}+u_{2i}\\ \psi_{i}=\exp\left(\omega_{0}+u_{3i}\right) \end{array}$$

$$u_{i}∼MVN\left(\left[\begin{array}{l} 0\\ 0\\ 0\\ 0 \end{array}\right],\left[\begin{array}{llll} \tau_{\mu } & & & \\ \tau_{21} & \tau_{\varphi } & & \\ \tau_{31} & \tau_{32} & \tau_{\beta t} & \\ \tau_{41} & \tau_{42} & \tau_{43} & \tau_{\psi } \end{array}\right]\right)$$

With covariates entering the model at Level 2 for the conditional model as follows:

Level 2:

(2) $$\begin{array}{l} \mu_{i}=\gamma_{00}+\gamma_{01}WM_{i}+\gamma_{02}Age_{i}+u_{0i}\\ \varphi_{i}=\gamma_{10}+\gamma_{11}WM_{i}+\gamma_{12}Age_{i}+u_{1i}\\ \beta_{ti}=\gamma_{20}+\gamma_{21}WM_{i}+\gamma_{22}Age_{i}+u_{2i}\\ \psi_{i}=\exp(\omega_{0}+\omega_{1}WM_{i}+w_{2}Age_{i}+u_{3i}) \end{array}$$

$$u_{i}∼MVN\left(\left[\begin{array}{l} 0\\ 0\\ 0\\ 0 \end{array}\right],\left[\begin{array}{llll} \tau_{\mu } & & & \\ \tau_{21} & \tau_{\varphi } & & \\ \tau_{31} & \tau_{32} & \tau_{\beta_{t}} & \\ \tau_{41} & \tau_{42} & \tau_{43} & \tau_{\psi } \end{array}\right]\right)$$

In the conditional model, the elements in ***u*_i_** are residual (co)variances. We can also represent this model graphically (Fig. 1*A*), where we can see that our four random effects reflect between-person differences in the within-person effects. For illustration purposes, we simulated exemplar time-series highlighting how high levels of a given random effect would change performance output (Fig. 1*B*).

The inclusion of age served as an important covariate to ensure that our results were not driven solely by systematic differences across age in mean RT (*r* = 0.242) and co-occurring age and WM FA (*r* = −0.470) across the lifespan. Because only a subset of participants who contributed data at Stage I (SRT) were invited to Stage II (VSTM and WM measures), we used a joint likelihood approach instead of the default conditional likelihood to allow missingness on exogenous variables without listwise deletion by estimating the mean and variance of each predictor (i.e., age and WM tract FA). The raw data are available on signing a data sharing request form (for more detail, see https://www.mrc-cbu.cam.ac.uk/datasets/camcan/). Analysis code and full model results are available on OSF (https://osf.io/nkjdt/).

## Results

### Unconditional model

We began by fitting two unconditional models to the SRT data: one with only fixed (i.e., zero variance) effects and the other with four random effects. The deviance information criteria (DIC_fixed_ = 1793, DIC_random_ = −44,107, ΔDIC = −45,900) overwhelmingly favored the inclusion of the four random effects (Asparouhov et al., 2018; McNeish and Hamaker, 2020). Fixed effects-only models in the VSTM data also showed very poor fit, bolstering the rationale for adopting a random effects approach to capture individual variability in the different components of the RT data. In a follow-up analyses, we compared the full random effect model to a set of models where we constrained each random effect to zero in turn. Model comparisons provided strong evidence in favor of including all random effects (ΔDICs ranging from −193 to −20,222). In other words, the model fit results suggested that the complexity of the behavioral data could not be appropriately captured by a single effect that held for all members of the sample and rather than allowing for individual differences in all four components of performance was necessary. For the SRT data, a model with four random effects showed that there is significant person-to-person variability in all four of the parameters modeled (Table 1). Examining the average within-person standardized effects (Schuurman et al., 2016), there was small positive inertia in participants' response time across adjacent trials (RT_t-1_; β = 0.036, *SD*_posterior_ = 0.003, 95% CI = [0.030, 0.042]) and participants got faster on average across the task (Trial_t_; β = −0.062, *SD*_posterior_ = 0.003, 95% CI = [−0.067, −0.056]). Results for the VSTM data confirmed this same general pattern.

**Table 1.** Model results for unconditional and full conditional model*^a^*

	Unconditional model				Full conditional model			
	Estimate	SD_post_	Lower	Upper	Estimate	SD_post_	Lower	Upper
Within-person standardized effects								
γ_10_	0.036	0.003	0.030	0.079	0.043	0.003	0.036	0.049
γ_20_	−0.053	0.003	−0.059	−0.047	−0.055	0.005	−0.063	−0.044
Between-person unstandardized effects								
γ_00_	−0.908	0.006	−0.920	−0.896	−0.495	0.253	−0.996	−0.008
γ_10_	0.036	0.004	0.029	0.043	0.196	0.128	−0.059	0.446
γ_20_	−0.001	0.000	−0.002	−0.001	−0.007	0.016	−0.041	0.022
ω_0_	−3.292	0.014	−3.309	−3.254	−1.689	0.594	−2.880	−0.536
τ_μ_	0.083	0.003	0.078	0.089	0.066	0.002	0.062	0.071
τ_φ_	0.008	0.001	0.006	0.010	0.013	0.001	0.011	0.015
$\tau_{\beta_{t}}$	1.9e-5	5.1e-7	1.8e-5	2.0e-5	1.7e-5	6.4e-7	1.5e-5	1.8e-5
τ_ψ_	0.424	0.014	0.398	0.452	0.356	0.013	0.330	0.382
Between-person standardized covariate effects								
ATR FA								
γ_01_					−0.149	0.049	−0.244	−0.051
γ_11_					−0.085	0.063	−0.209	0.040
γ_21_					0.042	0.074	−0.096	0.194
ω_1_					−0.197	0.051	−0.296	−0.095
Age								
γ_02_					0.351	0.032	0.287	0.413
γ_12_					0.012	0.047	−0.082	0.105
γ_22_					−0.072	0.045	−0.157	0.020
ω_2_					0.272	0.034	0.204	0.337
Variance explained (*R*^2^)								
log(RT_t-1_)	0.096	0.001	0.094	0.099	0.163	0.003	0.158	0.168
μ*_i_*					0.201	0.018	0.167	0.238
φ*_i_*					0.010	0.010	0.001	0.038
β*_ti_*					0.013	0.020	0.003	0.041
ψ*_i_*					0.169	0.020	0.133	0.212

*^a^*Estimate, Sample-recovered parameter; SD_post_, posterior SD; Lower, Upper, boundaries of the credible intervals.

**Figure 2.**
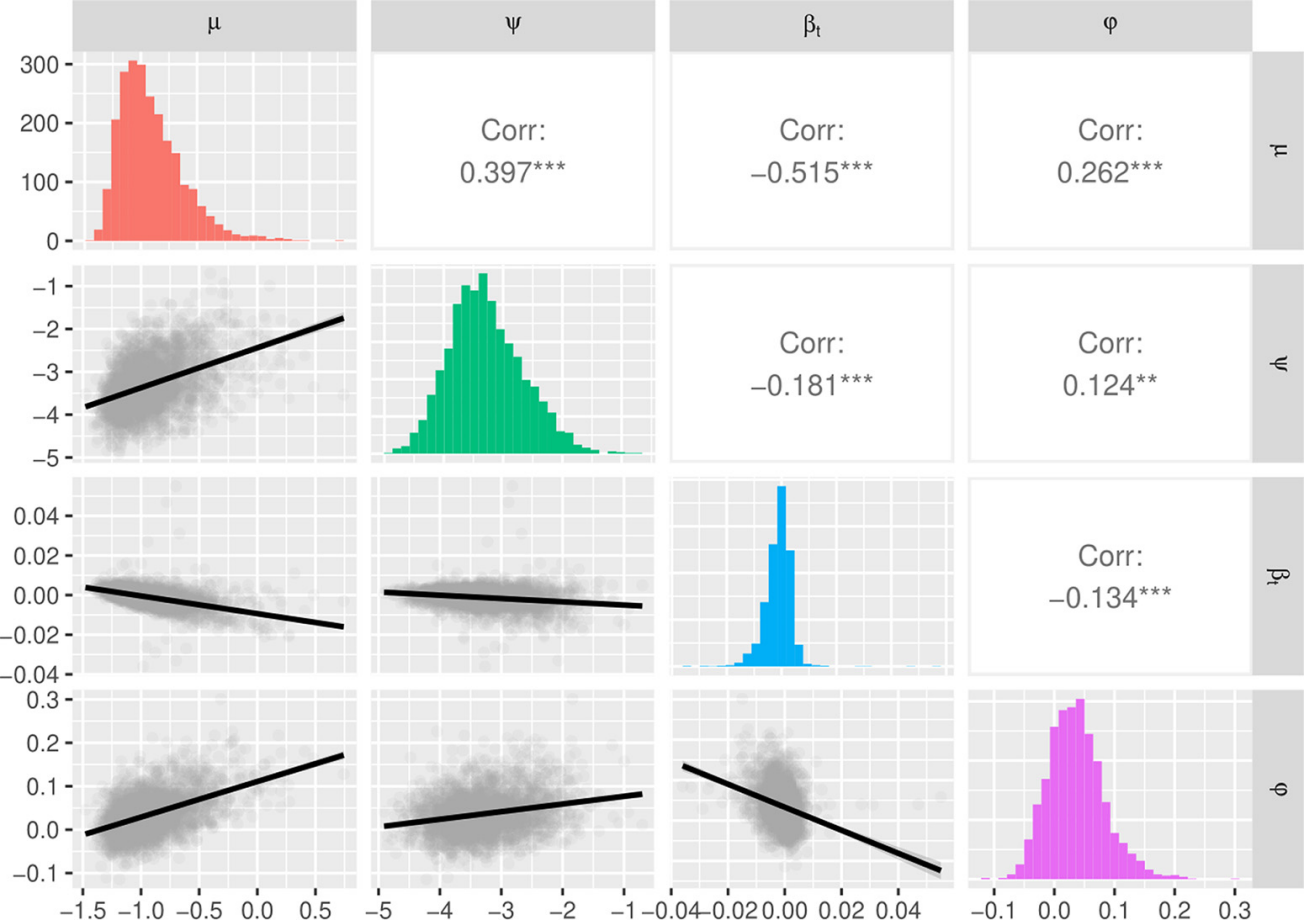
Distributions of random effects from the simple RT task. Each of the four random effects showed significant between-person variability. Mean performance (μ), variability (ψ), and inertia (φ; i.e., the autoregressive effect) were all positively correlated, and negatively correlated with the trend of performance across the task (β*_t_*).

When examining the pattern of correlations, there was some heterogeneity between the tasks. In the SRT data, the mean (μ), variability (ψ), and autoregressive (φ) random effects were all positively correlated with one another, and all negatively correlated with the trend effect (β*_t_*; Fig. 2). Of particular note was the correlation between mean RT and response variability which was moderately positive (*r* = 0.397, 95% CI = [0.359, 0.434]), suggesting that we could achieve separation of individuals' mean and variability in performance (compared with *r* = 0.666 between mean RT and a simple iSD measure). Interestingly, mean RT in the VSTM data showed a different pattern of relations (final response: *r* = −0.398, 95% CI = [−0.465, −0.328]), being negatively correlated with the other random effects. This perhaps indicates more of a ceiling effect, rather than the floor effect seen in simple speeded response tasks.

### Model based and observed measures of variability

**Figure 3.**
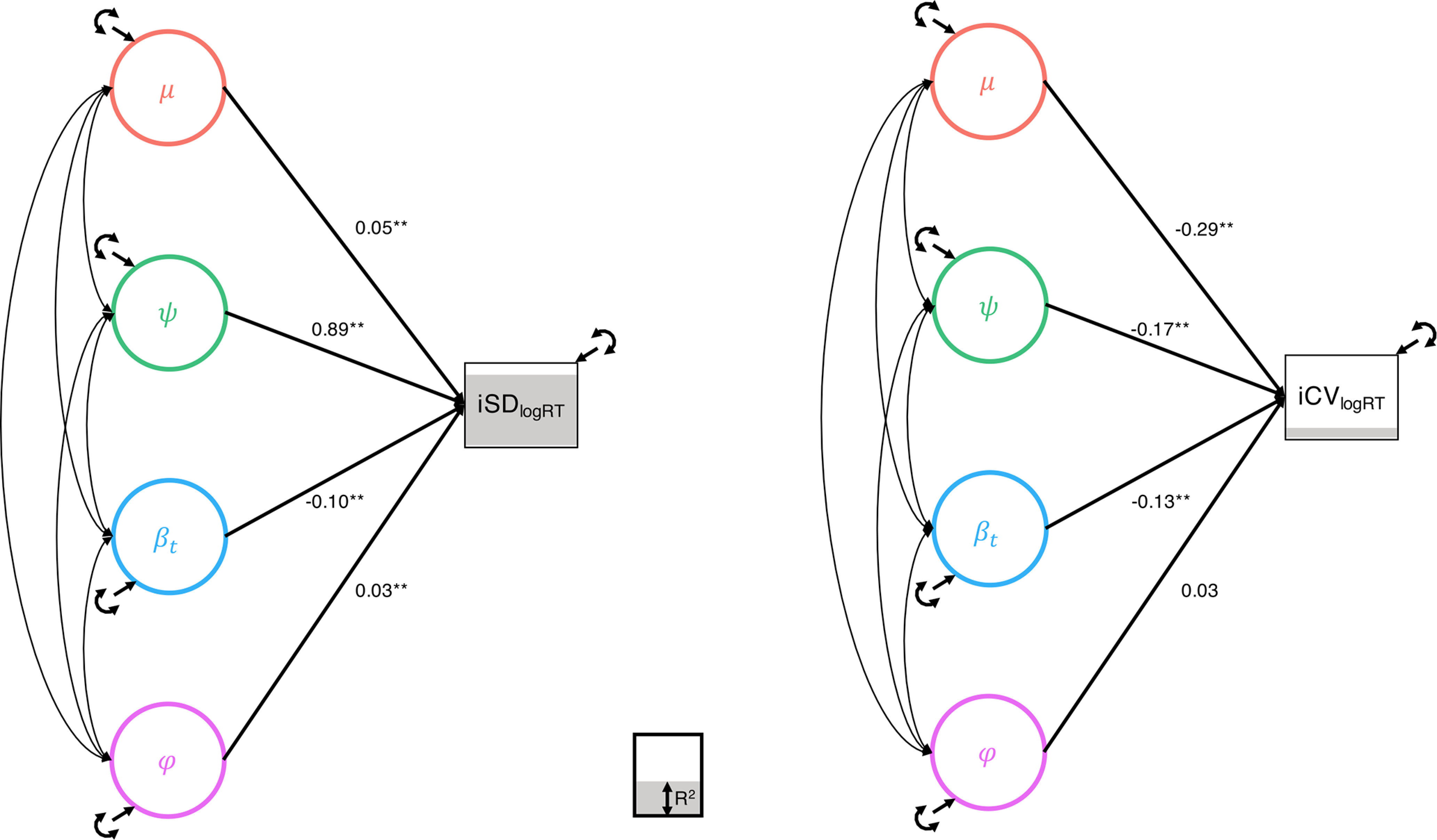
Explaining observed metrics of interindividual variability in SRT. While iSD was well described by the linear combination of the four random effects, the iCV showed surprising negative relationships with the metrics of mean and variability in performance derived from the DSEM. However, both metrics showed significant contamination by more than one random effect, especially mean performance (μ) and the trend (β*_t_*).

To further clarify the relationship between the DSEM-derived components of behavioral performance and traditional measures calculated directly from the data (iSD and iCV), we calculated these observed measures on the trial-level RT values after taking the natural log and entered the factor score estimates of the random effects as predictors in a multiple regression analysis (Fig. 3). The iSD measure was significantly predicted by all four random effects, while the iCV measure was significantly predicted by the mean, variability, and trend but not inertia factor scores. Interestingly, while variability strongly related to iSD (β = 0.885, *SE* = 0.007, *p* < 0.001), it weakly and negatively related to iCV (β = −0.167, *SE* = 0.021, *p* < 0.001). Indeed, iCV was most strongly related to mean performance (β = −0.293, *SE* = 0.027, *p* < 0.001) and the variance explained in iCV was relatively low (*R*^2^ = 0.111), especially compared with the high variance explained by the four random effects in iSD (*R*^2^ = 0.894). However, both analyses suggest that these observed metrics of intraindividual variability are significantly contaminated by the more complex components of performance, highlighting the utility of the DSEM approach.

### Conditional model without age

**Figure 4.**
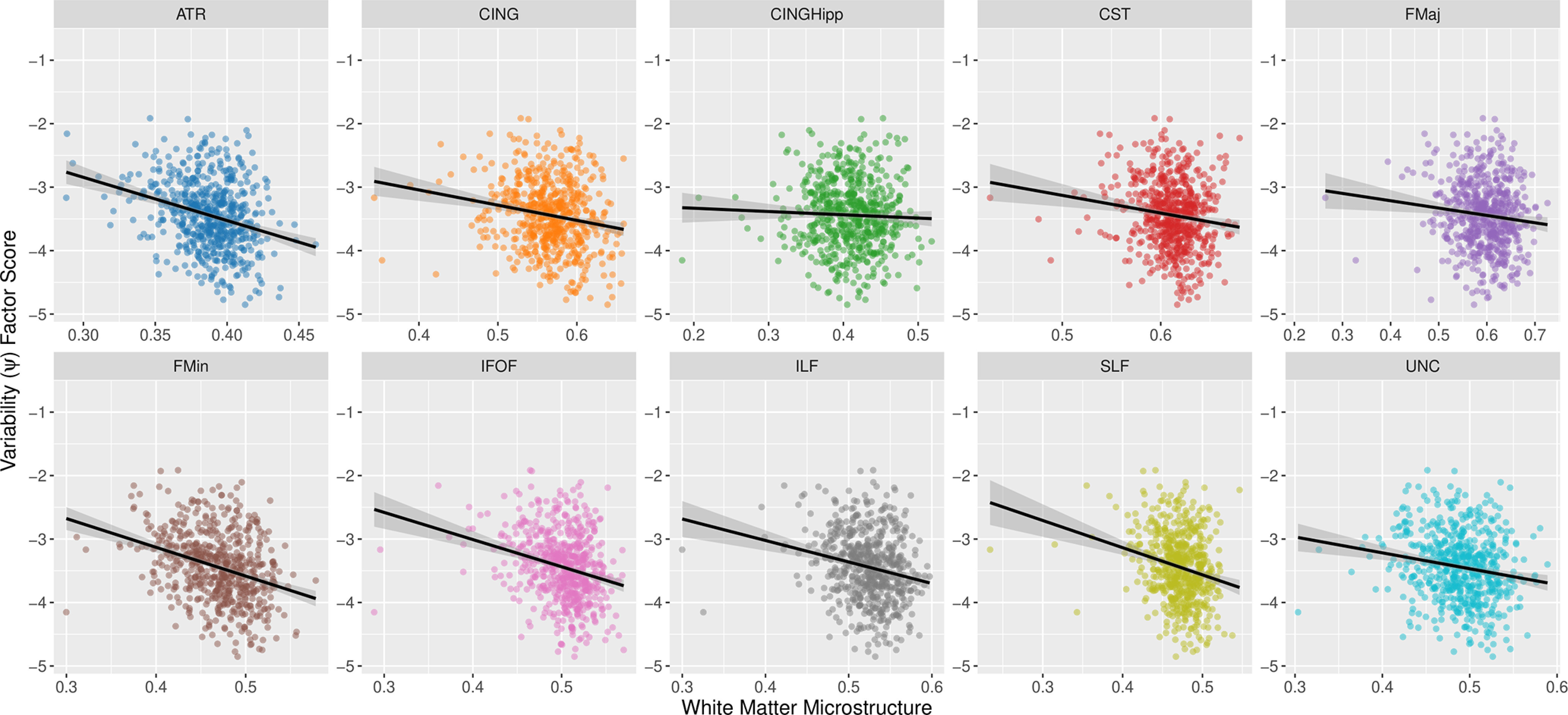
WM microstructure and variability. Across 9 of the 10 canonical tracts, reduced WM FA predicted more variability in response times (in the CINGHipp tract, there was no significant relationship).

We next included the 10 WM tracts as predictors in separate models at the between-person level predicting the random effects. As mentioned previously, we focus here on the model with the ATR but note any differences between models with other tracts (all models for the other tracts are available at https://osf.io/nkjdt/). As hypothesized, reduced WM FA predicted both slower response times (β = −0.322, *SD*_posterior_ = 0.042, 95% CI = [−0.401, −0.237]) and increased response variability (β = −0.344, *SD*_posterior_ = 0.042, 95% CI = [−0.423, −0.260]). Furthermore, lower WM FA predicted a higher autoregressive effect (β = −0.123, *SD*_posterior_ = 0.053, 95% CI = [−0.229, −0.021]) and a less positive trend (β = 0.119, *SD*_posterior_ = 0.034, 95% CI = [0.053, 0.186]). The addition of the ATR covariate reduced the residual correlation between mean RT and response variability (*r* = 0.263, 95% CI = [0.268, 0.371]). The other tracts had similar effects (although the effects for the CINGHipp were not significant) in the SRT data (Fig. 4). In the VSTM data, reduced ATR FA predicted only slower mean RT (β = −0.919, *SD*_posterior_ = 0.344, 95% CI = [−1.581, −0.239]) but not response variability (β = −0.653, *SD*_posterior_ = 0.787, 95% CI = [−2.198, 0.878].

### Conditional model with age

**Figure 5.**
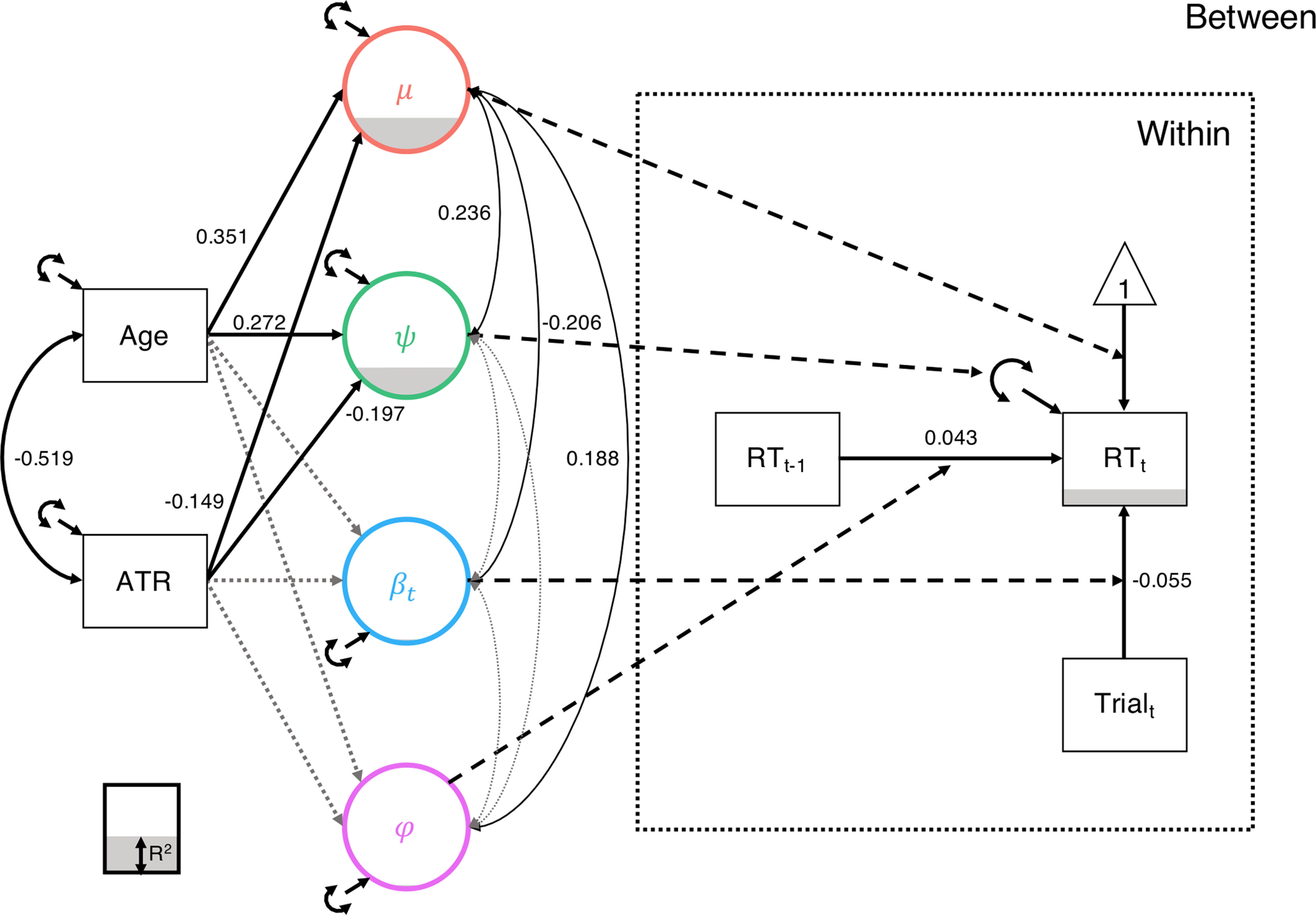
Conditional model of simple RT task with age and WM microstructure. Age and WM FA had opposite predictive effects, with older individuals showing slower and more variable responding, while those with greater WM FA showed the opposite pattern (i.e., faster, more consistent responding). Solid black paths represent significant regression effects (single-headed, directed paths; e.g., the effect of Trial_t_ on RT_t_) and covariances (double-headed, undirected paths; e.g., μ with ψ). Grey, short-dashed paths represent non-significant effects (e.g., the effect of ATR FA on φ). Black, long-dashed paths link random effects at the between-person level with their corresponding effect at the within person level. Explained variance (i.e., *R*^2^) is represented by the proportion of the outcome variable shaded in gray.

Finally, we included age as an additional covariate predicting the random effects to account for the likely confounding of systematic WM FA variation decreases across the adult lifespan (Fig. 1). Results suggested that older individuals showed slower average RT (β = 0.351, *SD*_posterior_ = 0.032, 95% CI = 0.287, 0.413]) and greater response variability (β = 0.272, *SD*_posterior_ = 0.034, 95% CI = 0.204, 0.337]; Table 1; see Fig. 5). Although somewhat attenuated compared with the conditional model without age, lower WM FA continued to uniquely predict slower mean performance (β = −0.149, *SD*_posterior_ = 0.049, 95% CI = [−0.244, −0.051]) and increased behavioral variability (β = −0.197, *SD*_posterior_ = 0.051, 95% CI = [−0.296, −0.095]). The inclusion of age and WM FA together explained 17%-20% of the between-person variance in mean RT (*R*^2^ = 0.201, *SD*_posterior_ = 0.018, 95% CI = [0.167, 0.238]) and RT variability (*R*^2^ = 0.169, *SD*_posterior_ = 0.020, 95% CI = [0.133, 0.212]), but only 1% of the variance in the trend across trials (*R*^2^ = 0.012, *SD*_posterior_ = 0.013, 95% CI = [0.003, 0.041]) and the variance in the autoregressive effect (*R*^2^ = 0.010, *SD*_posterior_ = 0.010, 95% CI = [0.001, 0.038]). At the within-person level, the lag-1 (i.e., autoregression) and trial (i.e., trend) effects accounted for 16% of the variance in individual trial RT (*R*^2^ = 0.163, *SD*_posterior_ = 0.003, 95% CI = [0.158, 0.168]).

## Discussion

In the modeling of human behavior, analysis of mean performance has preoccupied the majority of research focus. Here, we offer compelling evidence that variability in performance holds unique additional value for a broader understanding of human behavior. Crucially, to accurately isolate the mechanisms and consequences of this parameter as distinct from the mean requires appropriate quantitative tools. Using a DSEM (Asparouhov et al., 2018) approach, we fit a model that separates out four distinct components of human RT behavior: mean performance, variability in response times, linear trends across the task, and inertia (i.e., autoregression) in performance from trial to trial. In a large lifespan cohort (Shafto et al., 2014), we then used this model to examine the associations between these components of performance across aging and individual differences in WM microstructure (Fig. 4). Following from a “neural noise” hypothesis (Kail, 1997; Peters, 2009; Voytek et al., 2015), we posited that reduced WM microstructure would predict greater variability in behavioral performance, reflecting a less consistent signal-to-noise ratio in the propagation of speeded responses in the task (Peters, 2009; Kievit et al., 2016; Dave et al., 2018). We found evidence that poorer WM microstructure, measured through FA, predicted increased variability, as well as decreased mean level performance (Fig. 2), and these relationships held when controlling for age (Fig. 5). Overall, these results highlight the promise of approaches that directly incorporate variability as a parameter in the model, with potentially unique etiologic mechanisms and consequences.

### Modeling complex human behavior

One of the primary benefits of adopting a DSEM framework here is the ability to directly model within- and between-person features that are often treated as noise in simpler analyses (e.g., variability and trend), as well as complex temporal features of performance (e.g., autoregression) that would be invisible to those models. Doing so not only allows us to deconfound measures of mean performance and variability (de Haan-Rietdijk et al., 2016), but to model individual differences in all four components of performance that can show unique patterns of relationship with other individual differences measures. In each of the three tasks, model comparisons overwhelmingly favored models with random effects of the four components over a model with fixed effects, suggesting that human behavioral performance cannot be captured adequately by models that do not incorporate individual differences. Even in RT data, which often have a strong correlation between mean performance and variability because of floor effects on RT, DSEM was able to model individual differences in these components with reasonably low correlation (*r* = 0.397).

The DSEM approach to modeling variability also allowed us to explore the relationships between the random effects of the four behavioral components and observed measures of interindividual variability calculated directly from the trial-level data. We showed that the iSD measure was strongly related to variability (ψ) from the DSEM, but with significant contributions from the other random effects, while iCV was most strongly related to the mean performance factor (μ) and negatively related to both the mean and variability factors (Fig. 3). Together, these results highlight the strength of the DSEM to separate out these confounded factors of performance into unique factors and cautions us against using simple observed measures of variability without taking steps to ensure that they are not confounded by other sources of individual differences (e.g., differential trends or autoregression).

The DSEM framework presented here has the potential for many additional expansions that can help to broaden our understanding of the role that variability plays in a wide range of phenomena. Two such extensions include showing that individual differences in variability have unique consequences by including distal outcomes from longitudinal data into this model. Another exciting extension would be to incorporate the between-person level of the DSEM into a standard growth model (Hancock et al., 2001) to map trajectories of individual differences in mean performance and variability. This type of approach could help to disentangle within- from between-person differences in variability that we found across age in the current investigation. The flexibility of DSEM to incorporate these, and other, complex effects makes it a powerful tool for understanding the interplay between individual differences in mean performance and variability across the lifespan.

### Testing a neural noise hypothesis

Neural noise has been hypothesized to play a role in age-related changes in motor (Sosnoff and Newell, 2011; Voytek et al., 2015), sensory (Tran et al., 2020), and cognitive (Voytek et al., 2015; Pertermann et al., 2019) abilities. Neural noise has also been proposed as a mechanism in various non-normative states, such as ADHD (Hearne et al., 2021) and dyslexia (Frey et al., 2019). Common between these different theories is the idea that noisy firing in neural networks impairs consistent and efficacious behavior. Kail (1997) proposed that WM impairment offered a potential structural mechanism for increase in neural noise across aging, which we tested in the current study. WM has also been shown to be important for predicting response time variability associated with attentional problems in adolescence, providing additional evidence that this is a theoretically plausible target for investigation (Wiker et al., 2022).

We used the DSEM to test our hypothesis that reduced WM microstructure would predict less consistent behavioral performance. To do so, we entered each of the 10 WM tracts (Hua et al., 2008; Kievit et al., 2016) in separate models as a predictor of each of the four components of behavior. Prior work had shown consistent evidence that enhanced WM measures related to faster overall performance (Turken et al., 2008; Fjell et al., 2011) and observed measures of variability (Tamnes et al., 2012). Here, we confirm these prior findings, showing that WM FA predicts both slower mean RT performance and heightened variability in RT. These results held across the different WM tracts, with the exception of the CINGHipp, which showed a nonsignificant effect. WM FA did not predict individual differences in the trend or autoregressive effects, although we have much less power to detect effects in the autoregressive parameter as it depends on complex temporal information. While further work remains to incorporate direct measures of neural oscillations into this model, this provides consistent evidence with prior electrophysiological data where greater neural noise is related to poorer performance (Voytek et al., 2015; Dave et al., 2018; Tran et al., 2020), increases during aging (Voytek and Knight, 2015; Nobukawa et al., 2019), and is correlated with WM morphology (Smit et al., 2012; van Straaten et al., 2015).

To establish the specificity of these relationships, we introduced age into the model as an additional predictor to control for the systematic differences in WM FA and associated changes in speeded responses across the adult lifespan (Bartzokis et al., 2010). In this full conditional model, we show that the relationships between WM and the mean and variability factors are preserved, although somewhat attenuated. Consistent with prior work, increased age and reduced WM FA were associated with poorer (i.e., slower and more variable) performance (Fig. 5). In sum, these results provide additional support for the neural noise hypothesis (Kail, 1997), reflecting a role for WM in the signal propagation needed for consistent performance and performance deficits when these fibers are compromised.

### Directions for future research

While there are many strengths to the current approach taken here, there are several avenues for further testing of the neural noise hypothesis that should be addressed in future research. First, longitudinal measures of WM tract and behavioral change over time would allow us to assess within-person dynamic relationships across aging. Additionally, while we tested the relationship between structure and behavioral output variability, an important follow-up test of the neural noise hypothesis would be to link structural brain changes during aging to evoked neural responses during behavior. Combining functional recordings, either fMRI (Garrett et al., 2021) or electrophysiological (McIntosh et al., 2008; Woolnough et al., 2022), with structural measures (e.g., Hearne et al., 2021) would shed important light onto the consequences of WM loss for function and behavior. One strength of the DSEM approach would be to combine time-series models for both recordings and behavior and link them at the factor level to measurements of structural WM, or to important cognitive outcomes across aging.

When studying the broader construct of variability, it is important to note that the adaptive versus maladaptive nature of variability is context-dependent. In the current work, variability of speeded responses on a relatively simple RT appears to reflect performance deficits. However, in other contexts, such as learning (McCormick and Telzer, 2017) or more complex tasks (McIntosh et al., 2008; Garrett et al., 2011), variability may allow for greater flexibility in representing the task space. Understanding when variability is related to improvements versus declines in cognitive performance will offer greater insights into the mechanistic processes underlying lifespan brain development.

In conclusion, the study of variability as an important marker of individual differences in behavior and cognition is still in the early phases of development. We presented here a powerful framework for modeling variability in concert with other components of behavior, including mean performance, trends over time, and inertia in trial-level performance. We further show that these individual differences can be used to test a theory of neural noise by including person-level predictors and found that both reduced WM microstructure and increased age uniquely predict poorer task performance, consistent with the importance of intact WM tracts for appropriate signal propagation. The results here highlight the promise of leveraging advanced behavioral modeling to move beyond a sole focus on mean differences and recognize variability as an important individual difference marker for understanding human behavior. By combining these models with measures of brain structure and function, this framework can be used to test a wide array of hypotheses for how these individual differences in variability emerge.

## References

[B1] Adam KC, Mance I, Fukuda K, Vogel EK (2015) The contribution of attentional lapses to individual differences in visual working memory capacity. J Cogn Neurosci 27:1601–1616. 10.1162/jocn_a_00811 25811710 PMC4494675

[B2] Asparouhov T, Hamaker EL, Muthén B (2018) Dynamic structural equation models. Struct Equation Modeling 25:359–388. 10.1080/10705511.2017.1406803 29624092

[B3] Bartzokis G, Lu PH, Tingus K, Mendez MF, Richard A, Peters DG, Oluwadara B, Barrall KA, Finn JP, Villablanca P, Thompson PM, Mintz J (2010) Lifespan trajectory of myelin integrity and maximum motor speed. Neurobiol Aging 31:1554–1562. 10.1016/j.neurobiolaging.2008.08.015 18926601 PMC2888859

[B4] Bennett IJ, Madden DJ (2014) Disconnected aging: cerebral white matter integrity and age-related differences in cognition. Neuroscience 276:187–205. 10.1016/j.neuroscience.2013.11.026 24280637 PMC4032380

[B5] Britton TC, Meyer BU, Benecke R (1991) Variability of cortically evoked motor responses in multiple sclerosis. Electroencephalogr Clin Neurophysiol 81:186–194. 10.1016/0168-5597(91)90071-5 1710967

[B6] Bunce D, Anstey K, Christensen H, Dear K, Wen W, Sachdev P (2007) White matter hyperintensities and within-person variability in community-dwelling adults aged 60–64 years. Neuropsychologia 45:2009–2015. 10.1016/j.neuropsychologia.2007.02.006 17382358

[B7] Dave S, Brothers TA, Swaab TY (2018) 1/f neural noise and electrophysiological indices of contextual prediction in aging. Brain Res 1691:34–43. 10.1016/j.brainres.2018.04.007 29679544 PMC5965691

[B8] de Haan-Rietdijk S, Kuppens P, Hamaker EL (2016) What's in a day? A guide to decomposing the variance in intensive longitudinal data. Front Psychol 7:891. 10.3389/fpsyg.2016.00891 27378986 PMC4906027

[B9] de Mooij SM, Henson RN, Waldorp LJ, Kievit RA (2018) Age differentiation within gray matter, white matter, and between memory and white matter in an adult life span cohort. J Neurosci 38:5826–5836. 10.1523/JNEUROSCI.1627-17.2018 29848485 PMC6010564

[B10] Dykiert D, Der G, Starr JM, Deary IJ (2012) Age differences in intra-individual variability in simple and choice reaction time: systematic review and meta-analysis. PLoS One 7:e45759. 10.1371/journal.pone.0045759 23071524 PMC3469552

[B11] Eizenman DR, Nesselroade JR, Featherman DL, Rowe JW (1997) Intraindividual variability in perceived control in an older sample: the MacArthur successful aging studies. Psychol Aging 12:489–502. 10.1037//0882-7974.12.3.489 9308096

[B12] Fiske DW, Rice L (1955) Intra-individual response variability. Psychol Bull 52:217–250. 10.1037/h0045276 14371891

[B13] Fjell AM, Westlye LT, Amlien IK, Walhovd KB (2011) Reduced white matter integrity is related to cognitive instability. J Neurosci 31:18060–18072. 10.1523/JNEUROSCI.4735-11.2011 22159119 PMC6634144

[B14] Folstein MF, Folstein SE, McHugh PR (1975) Mini-mental state. J Psychiatr Res 12:189–198. 10.1016/0022-3956(75)90026-6 1202204

[B15] Frey A, François C, Chobert J, Besson M, Ziegler JC (2019) Behavioral and electrophysiological investigation of speech perception deficits in silence, noise and envelope conditions in developmental dyslexia. Neuropsychologia 130:3–12. 10.1016/j.neuropsychologia.2018.07.033 30075216

[B16] Gamaldo AA, An Y, Allaire JC, Kitner-Triolo MH, Zonderman AB (2012) Variability in performance: identifying early signs of future cognitive impairment. Neuropsychology 26:534–540. 10.1037/a0028686 22746310 PMC3530416

[B17] Garrett DD, Kovacevic N, McIntosh AR, Grady CL (2011) The importance of being variable. J Neurosci 31:4496–4503. 10.1523/JNEUROSCI.5641-10.2011 21430150 PMC3104038

[B18] Garrett DD, Skowron A, Wiegert S, Adolf J, Dahle CL, Lindenberger U, Raz N (2021) Lost dynamics and the dynamics of loss: longitudinal compression of brain signal variability is coupled with declines in functional integration and cognitive performance. Cereb Cortex 31:5239–5252. 10.1093/cercor/bhab154 34297815 PMC8491679

[B19] Gelman A, Rubin DB (1992) Inference from iterative simulation using multiple sequences. Statist Sci 7:457–472 10.1214/ss/1177011136

[B20] Halliday DW, Gawryluk JR, Garcia-Barrera MA, MacDonald SW (2019) White matter integrity is associated with intraindividual variability in neuropsychological test performance in healthy older adults. Front Hum Neurosci 13:352. 10.3389/fnhum.2019.00352 31680907 PMC6803513

[B21] Hancock GR, Kuo WL, Lawrence FR (2001) An illustration of second-order latent growth models. Structural Equation Modeling 8:470–489. 10.1207/S15328007SEM0803_7

[B22] Haynes BI, Bauermeister S, Bunce D (2017) A systematic review of longitudinal associations between reaction time intraindividual variability and age-related cognitive decline or impairment, dementia, and mortality. J Int Neuropsychol Soc 23:431–445. 10.1017/S1355617717000236 28462758

[B23] Hearne LJ, Lin HY, Sanz-Leon P, Tseng WY, Gau SS, Roberts JA, Cocchi L (2021) ADHD symptoms map onto noise-driven structure-function decoupling between hub and peripheral brain regions. Mol Psychiatry 26:4036–4045. 10.1038/s41380-019-0554-6 31666679

[B24] Hua K, Zhang J, Wakana S, Jiang H, Li X, Reich DS, Calabresi PA, Pekar JJ, van Zijl PC, Mori S (2008) Tract probability maps in stereotaxic spaces: analyses of white matter anatomy and tract-specific quantification. Neuroimage 39:336–347. 10.1016/j.neuroimage.2007.07.053 17931890 PMC2724595

[B25] Jones DK, Knösche TR, Turner R (2013) White matter integrity, fiber count, and other fallacies: the do's and don'ts of diffusion MRI. Neuroimage 73:239–254. 10.1016/j.neuroimage.2012.06.081 22846632

[B26] Kail R (1997) The neural noise hypothesis: evidence from processing speed in adults with multiple sclerosis. Aging Neuropsychol Cogn 4:157–165. 10.1080/13825589708256644

[B27] Kievit RA, Davis SW, Griffiths J, Correia MM, Cambridge Centre for Ageing and Neuroscience, Henson RN (2016) A watershed model of individual differences in fluid intelligence. Neuropsychologia 91:186–198. 10.1016/j.neuropsychologia.2016.08.008 27520470 PMC5081064

[B28] Lavery MR, Acharya P, Sivo SA, Xu L (2019) Number of predictors and multicollinearity: what are their effects on error and bias in regression? Commun Stat 48:27–38. 10.1080/03610918.2017.1371750

[B29] Li SC, Lindenberger U (1999) Cross-level unification: a computational exploration of the link between deterioration of neurotransmitter systems and dedifferentiation of cognitive abilities in old age. In: Cognitive neuroscience of memory, pp 103–146. Göttingen, Germany: Hogrefe and Huber.

[B30] Li SC, Lindenberger U, Hommel B, Aschersleben G, Prinz W, Baltes PB (2004) Transformations in the couplings among intellectual abilities and constituent cognitive processes across the life span. Psychol Sci 15:155–163. 10.1111/j.0956-7976.2004.01503003.x 15016286

[B31] MacDonald SW, Nyberg L, Bäckman L (2006) Intra-individual variability in behavior: links to brain structure, neurotransmission and neuronal activity. Trends Neurosci 29:474–480. 10.1016/j.tins.2006.06.011 16820224

[B32] McCormick EM (2021) Multi-level multi-growth models: new opportunities for addressing developmental theory using advanced longitudinal designs with planned missingness. Dev Cogn Neurosci 51:101001. 10.1016/j.dcn.2021.101001 34391004 PMC8363832

[B33] McCormick EM, Telzer EH (2017) Adaptive adolescent flexibility: neurodevelopment of decision-making and learning in a risky context. J Cogn Neurosci 29:413–423. 10.1162/jocn_a_01061 28129057 PMC5362273

[B34] McIntosh AR, Kovacevic N, Itier RJ (2008) Increased brain signal variability accompanies lower behavioral variability in development. PLoS Comput Biol 4:e1000106. 10.1371/journal.pcbi.1000106 18604265 PMC2429973

[B35] McNeish D, Hamaker EL (2020) A primer on two-level dynamic structural equation models for intensive longitudinal data in Mplus. Psychol Methods 25:610–635. 10.1037/met0000250 31855015

[B36] Nesselroade J (1991) The warp and woof of the developmental fabric. In: Visions of aesthetics, the environment & development: The legacy of Joachim F. Wohlwill (Downs RM, Liben LS, Palermo DS eds), pp 213–240. Lawrence Erlbaum Associates, Inc.

[B37] Nobukawa S, Kikuchi M, Takahashi T (2019) Changes in functional connectivity dynamics with aging: a dynamical phase synchronization approach. Neuroimage 188:357–368. 10.1016/j.neuroimage.2018.12.008 30529509

[B38] Pertermann M, Mückschel M, Adelhöfer N, Ziemssen T, Beste C (2019) On the interrelation of 1/f neural noise and norepinephrine system activity during motor response inhibition. J Neurophysiol 121:1633–1643. 10.1152/jn.00701.2018 30811254

[B39] Peters A (2009) The effects of normal aging on myelinated nerve fibers in monkey central nervous system. Front Neuroanat 3:11. 10.3389/neuro.05.011.2009 19636385 PMC2713738

[B40] Salthouse TA (1996) The processing-speed theory of adult age differences in cognition. Psychol Rev 103:403–428. 10.1037/0033-295x.103.3.403 8759042

[B41] Schuurman NK, Ferrer E, de Boer-Sonnenschein M, Hamaker EL (2016) How to compare cross-lagged associations in a multilevel autoregressive model. Psychol Methods 21:206–221. 10.1037/met0000062 27045851

[B42] Shafto MA, Tyler LK, Dixon M, Taylor JR, Rowe JB, Cusack R, Calder AJ, Marslen-Wilson WD, Duncan J, Dalgleish T, Henson RN, Brayne C, Matthews FE, Cambridge Centre for Ageing and Neuroscience (2014) The Cambridge Centre for Ageing and Neuroscience (Cam-CAN) study protocol: a cross-sectional, lifespan, multidisciplinary examination of healthy cognitive ageing. BMC Neurol 14:204. 10.1186/s12883-014-0204-1 25412575 PMC4219118

[B43] Smit DJ, Boersma M, Schnack HG, Micheloyannis S, Boomsma DI, Pol HE, Stam CJ, Geus E (2012) The brain matures with stronger functional connectivity and decreased randomness of its network. PLoS One 7:e36896. 10.1371/journal.pone.0036896 22615837 PMC3352942

[B44] Sorg SF, Merritt VC, Clark AL, Werhane ML, Holiday KA, Schiehser DM, Bondi M, Delano-Wood L (2021) Elevated intraindividual variability in executive functions and associations with white matter microstructure in veterans with mild traumatic brain injury. J Int Neuropsychol Soc 27:305–314. 10.1017/S1355617720000879 32967755 PMC8462939

[B45] Sosnoff JJ, Newell KM (2011) Aging and motor variability: a test of the neural noise hypothesis. Exp Aging Res 37:377–397. 10.1080/0361073X.2011.590754 21800971

[B46] Tamnes CK, Fjell AM, Westlye LT, Ostby Y, Walhovd KB (2012) Becoming consistent: developmental reductions in intraindividual variability in reaction time are related to white matter integrity. J Neurosci 32:972–982. 10.1523/JNEUROSCI.4779-11.2012 22262895 PMC6621149

[B47] Taylor JR, Williams N, Cusack R, Auer T, Shafto MA, Dixon M, Tyler LK, Cam-CAN, Henson RN (2017) The Cambridge Centre for Ageing and Neuroscience (Cam-CAN) data repository: structural and functional MRI, MEG, and cognitive data from a cross-sectional adult lifespan sample. Neuroimage 144:262–269. 10.1016/j.neuroimage.2015.09.018 26375206 PMC5182075

[B48] Tran TT, Rolle CE, Gazzaley A, Voytek B (2020) Linked sources of neural noise contribute to age-related cognitive decline. J Cogn Neurosci 32:1813–1822. 10.1162/jocn_a_01584 32427069 PMC7474516

[B49] Turken U, Whitfield-Gabrieli S, Bammer R, Baldo JV, Dronkers NF, Gabrieli JD (2008) Cognitive processing speed and the structure of white matter pathways: convergent evidence from normal variation and lesion studies. Neuroimage 42:1032–1044. 10.1016/j.neuroimage.2008.03.057 18602840 PMC2630965

[B50] van Straaten EC, den Haan J, de Waal H, van der Flier WM, Barkhof F, Prins ND, Stam CJ (2015) Disturbed phase relations in white matter hyperintensity based vascular dementia: an EEG directed connectivity study. Clin Neurophysiol 126:497–504. 10.1016/j.clinph.2014.05.018 24969377

[B51] Voytek B, Knight RT (2015) Dynamic network communication as a unifying neural basis for cognition, development, aging, and disease. Biol Psychiatry 77:1089–1097. 10.1016/j.biopsych.2015.04.016 26005114 PMC4443259

[B52] Voytek B, Kramer MA, Case J, Lepage KQ, Tempesta ZR, Knight RT, Gazzaley A (2015) Age-related changes in 1/f neural electrophysiological noise. J Neurosci 35:13257–13265. 10.1523/JNEUROSCI.2332-14.2015 26400953 PMC4579381

[B53] Wang L, Grimm KJ (2012) Investigating reliabilities of intraindividual variability indicators. Multivariate Behav Res 47:771–802. 10.1080/00273171.2012.715842 26754444

[B54] Wiker T, Norbom LB, Beck D, Agartz I, Andreassen OA, Alnæs D, Dahl A, Eilertsen E, Moberget T, Ystrøm E, Westlye LT, Lebel C, Huster RJ, Tamnes CK (2022) Reaction time variability in children is specifically associated with attention problems and regional white matter microstructure. PsyArXiv. 10.31234/osf.io/pyr65. 37003411

[B55] Williams BR, Hultsch DF, Strauss EH, Hunter MA, Tannock R (2005) Inconsistency in reaction time across the life span. Neuropsychology 19:88–96. 10.1037/0894-4105.19.1.88 15656766

[B56] Woodrow H (1932) Quotidian variability. Psychol Rev 39:245–256. 10.1037/h0073076

[B57] Woolnough O, Kadipasaoglu CM, Conner CR, Forseth KJ, Rollo PS, Rollo MJ, Baboyan VG, Tandon N (2022) Dataset of human intracranial recordings during famous landmark identification. Sci Data 9:28. 10.1038/s41597-022-01125-8 35102154 PMC8803828

